# Transcription of endogenous retroviruses in senescent cells contributes to the accumulation of double-stranded RNAs that trigger an anti-viral response that reinforces senescence

**DOI:** 10.1038/s41419-024-06548-2

**Published:** 2024-02-21

**Authors:** Eros Di Giorgio, Liliana Ranzino, Vanessa Tolotto, Emiliano Dalla, Matteo Burelli, Nicolò Gualandi, Claudio Brancolini

**Affiliations:** 1 Laboratory of Biochemistry, Department of Medicine, Università degli Studi di Udine, p.le Kolbe 4, 33100 Udine, Italy; 2 Laboratory of Epigenomics, Department of Medicine, Università degli Studi di Udine, p.le Kolbe 4, 33100 Udine, Italy

**Keywords:** Senescence, Epigenetics

## Abstract

An important epigenetic switch marks the onset and maintenance of senescence. This allows transcription of the genetic programs that arrest the cell cycle and alter the microenvironment. Transcription of endogenous retroviruses (ERVs) is also a consequence of this epigenetic switch. In this manuscript, we have identified a group of ERVs that are epigenetically silenced in proliferating cells but are upregulated during replicative senescence or during various forms of oncogene-induced senescence, by RAS and Akt, or after HDAC4 depletion. In a HDAC4 model of senescence, removal of the repressive histone mark H3K27me3 is the plausible mechanism that allows the transcription of intergenic ERVs during senescence. We have shown that ERVs contribute to the accumulation of dsRNAs in senescence, which can initiate the antiviral response via the IFIH1-MAVS signaling pathway and thus contribute to the maintenance of senescence. This pathway, and MAVS in particular, plays an active role in shaping the microenvironment and maintaining growth arrest, two essential features of the senescence program.

## Introduction

Cellular senescence is controlled by a complex genetic program, aimed at achieving permanent growth arrest and altering the microenvironment [1]. Once established, cellular senescence contributes to aging and progressive dysfunctions of tissues and organs [2, 3]. Senescent cells secrete various cytokines and modulators of the ECM (extracellular matrix) defined as SASP (senescence associated secretory phenotype). SASP exerts several effects, including reinforcing senescence and activating the immune clearance of senescent cells. SASP may also promote proliferation of adjacent cells and stem cell maintenance to support tissue regeneration [4, 5].

To fulfill the genetic program of senescence, cells undergo timely accurate epigenetic resetting [6]. This reorganization involves the restructuring of proximal and distal regulatory elements (promoters and enhancers) with the stabilization of new super-enhancers (SEs) [7–9].

Transposable elements make up nearly 50% of the human genome, and about 8% of them are endogenous retroviruses (ERVs) [10, 11]. ERVs are retrotransposons that contain long-terminal-repeats (LTRs). ERVs are normally inactive but under conditions such as DNA damage, viral infection or aging, they become activated [12, 13]. ERVs expression is normally silenced by epigenetic mechanisms that maintain a repressed chromatin state. DNA hypomethylating agents and HDACs inhibitors can upregulate ERVs expression [14–16].

HDACs may be part of repressive complexes responsible for silencing proviruses, also involved in regulating senescence [17–22]. Notably, the class IIa HDAC family member HDAC4, acts as a brake on senescence by supervising H3K27 acetylation at specific SEs. During senescence, HDAC4 is degraded by the ubiquitin-proteasome system in a GSK3β-dependent manner [8]. This epigenetic switch could contribute to the resurgence of certain ERVs. Indeed, previous studies have shown that epigenetic perturbations elicit the transcription of ERVs during cellular senescence in aging and cancer [15, 23–25]. In this manuscript, we investigated whether ERVs are regulated during different forms of senescence and what role these retroviral elements might play in the senescence program.

## Results

### Epigenetic resetting and upregulation of ERVs transcription during senescence

We hypothesized that the epigenetic perturbations associated with senescence might also be involved in the upregulation of some ERVs. To investigate this hypothesis, we selected a set of ERVs (*ERV9-1, ERVW-1, ERVFC1-1, ERVH48-1*, Table S1), that are upregulated after pharmacologically induced epigenetic switch [15]. These ERVs are good candidates for upregulation during the epigenetic switch that characterizes senescence [6].

**Fig. 1:**
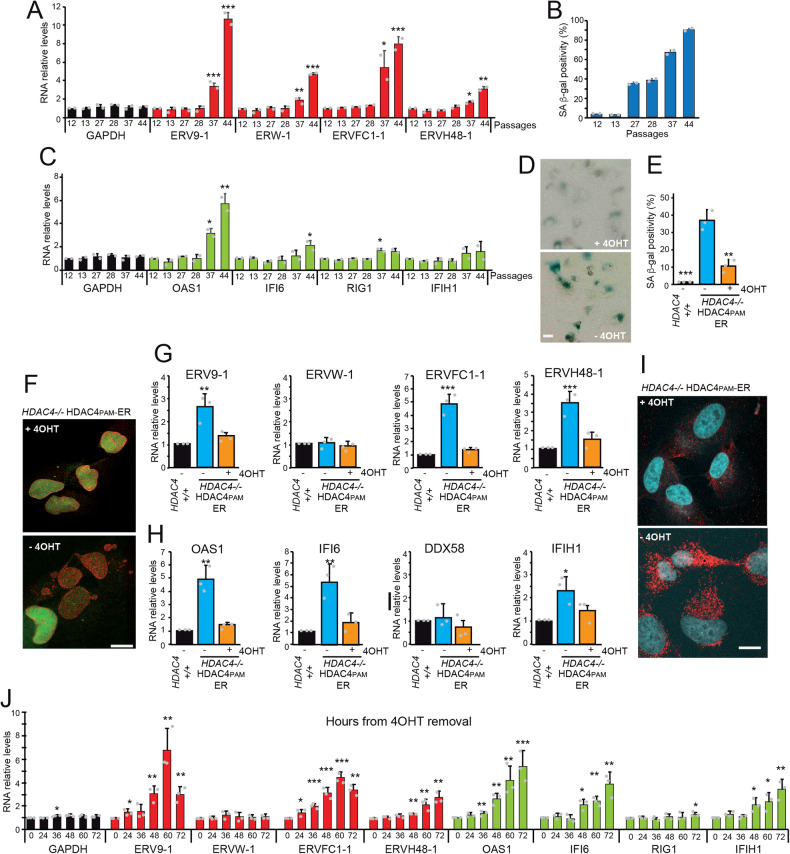
Upregulation of ERVs expression in different models of senescence. **A** Expression levels of the indicated ERVs in IMR90 cells undergoing replicative senescence. RNAs were extracted at the indicated passages of cultured cells and processed for qRT-PCR. Data are relative to passage 12. Mean ± SD; *n* = 2. *p < 0.05, **p < 0.01, ***p < 0.001, *t*-test relative to PD12. **B** SA β-gal positivity in IMR90 cells undergoing replicative senescence. Cells were fixed processed for SA β-gal assay and scored at the microscope. **C** Expression levels of the indicated ISGs in IMR90 cells undergoing replicative senescence. RNAs were extracted at the indicated passages of cultured cells and processed for qRT-PCR. Data are relative to passage 12. Mean ± SD; *n* = 2. *p < 0.05, **p < 0.01, ***p < 0.001, *t*-test relative to PD12. **D** SA β-gal positivity in SK-LMS-1 cells knocked-out for HDAC4 and re-expressing a 4OHT inducible, PAM mutated version of HDAC4 (SK-LMS-1^*HDAC4-/-/HDAC4PAM-ER*^). Bar = 50 µm. **E** Quantitative analysis of SA-β-gal positivity in SK-LMS-1^*HDAC4-/-/HDAC4PAM-ER*^ grown in the presence or not of 4OHT. Mean ± SD; *n* = 3. **p < 0.01, ***p < 0.001, *t*-test relative to -4OHT condition. **F** Immunofluorescence analysis of LMNA (red) and H1.2-GFP (green) distribution in SK-LMS-1^*HDAC4-/-/HDAC4PAM-ER*^ grown of not in the presence of 4OHT for 48 h. Confocal images are shown in pseudocolors. Bar = 10 µm. **G** Expression levels of the indicated ERVs in SK-LMS-1 cells and SK-LMS-1^*HDAC4-/-/HDAC4PAM-ER*^ grown in the presence or not of 4OHT. Mean ± SD; *n* = 3. **p < 0.01, ***p < 0.001, *t*-test relative to -4OHT condition. **H** Expression levels of the indicated ISGs in SK-LMS-1 cells and SK-LMS-1^*HDAC4-/-/HDAC4PAM-ER*^ grown in the presence or not of 4OHT. Mean ± SD; *n* = 3. *p < 0.05, **p < 0.01, *t*-test relative to -4OHT condition. **I** Representative confocal pictures of SK-LMS-1^*HDAC4-/-/HDAC4PAM-ER*^ grown for 48 h in the presence or not of 4OHT. Immunofluorescence was performed to visualize dsRNAs (red) and nuclei (blue). Confocal images are shown in pseudocolors. Bar 10 µM. **J** Expression levels of the indicated ERVs and ISGs in SK-LMS-1^*HDAC4-/-/HDAC4PAM-ER*^ cells grown in the absence of 4OHT for the indicate hours. Mean ± SD; *n* = 3. *p < 0.05, **p < 0.01, *t*-test relative to time zero.

First, we examined the expression levels of these ERVs during replicative senescence. IMR90 diploid fibroblasts harvested at increasing population doubling were chosen as replicative senescent model [8]. All four ERVs were upregulated during replicative senescence (Fig. 1A). The SA β-gal staining confirmed the induction of senescence (Fig. 1B). Transcriptional activation and chromatin remodeling at specific ERV sequences during senescence is coupled to upregulation of the interferon (IFN) response [26]. More generally, reactivation of ERVs can trigger the innate immune response [27, 28]. Induction of the IFN response during replicative senescence has been studied using a signature of IFN-stimulated genes (ISGs): *OAS1, IFI6, RIG1, IFIH1*. The upregulation of *OAS1, IFI6, IFIH1*, and *RIG1* was coupled to the induction of ERVs expression (Fig. 1C).

As a second model of cellular senescence, we chose low-grade leiomyosarcoma cells SK-LMS-1 that undergo senescence after HDAC4 knock-out. In SK-LMS-1 cells, HDAC4 is required to suppress the activity of typical enhancers (TEs) and SEs that control the senescence program [8]. Therefore, we could refer to this model as epigenetically induced senescence. SK-LMS-1^*HDAC4-/-/HDAC4PAM-ER*^, in which HDAC4 is knocked-out and which re-expresses a PAM mutant inducible version of HDAC4 fused to ER (estrogen receptor), was selected as an inducible model for senescence [8]. In this cell line, HDAC4 can be turned on and off by removing tamoxifen (4OHT) and senescence induced, as proved by SA β-gal staining (Fig. 1D, E) and Lamin B1 downregulation (Fig. S1A).

Histone H1-mediated heterochromatinization is important to silence noncoding RNA derived from heterochromatic repeats or endogenous retroviruses to avoid a nonspecific IFN response [29]. Abrogation of HDAC4 expression after removal of 4OHT leads to downregulation of H1 (Fig. 1F), upregulation of ERVs, except *ERVW-1*, which contains a coding gene (syncytin1) (Fig. 1G), and IFN genes (Fig. 1H). Immunofluorescence analyses using the J2 antibody [30] demonstrate the accumulation of dsRNAs species in SK-LMS-1^*HDAC4-/-/HDAC4PAM-ER*^ cells after depletion of HDAC4 (Fig. 1I).

To better capture the timing of activation of ERVs expression and IFN response, we performed a time-course analysis. Samples were collected at different time points after removal of 4OHT. Figure 1J shows that induction of ERVs and upregulation of IFN genes are time-coupled responses.

In summary, these results show that epigenetically regulated ERVs are induced in two different models of senescence.

### Senescence induced after HDAC4 knock-out elicits the expression of ERVs and of the IFN response

**Fig. 2:**
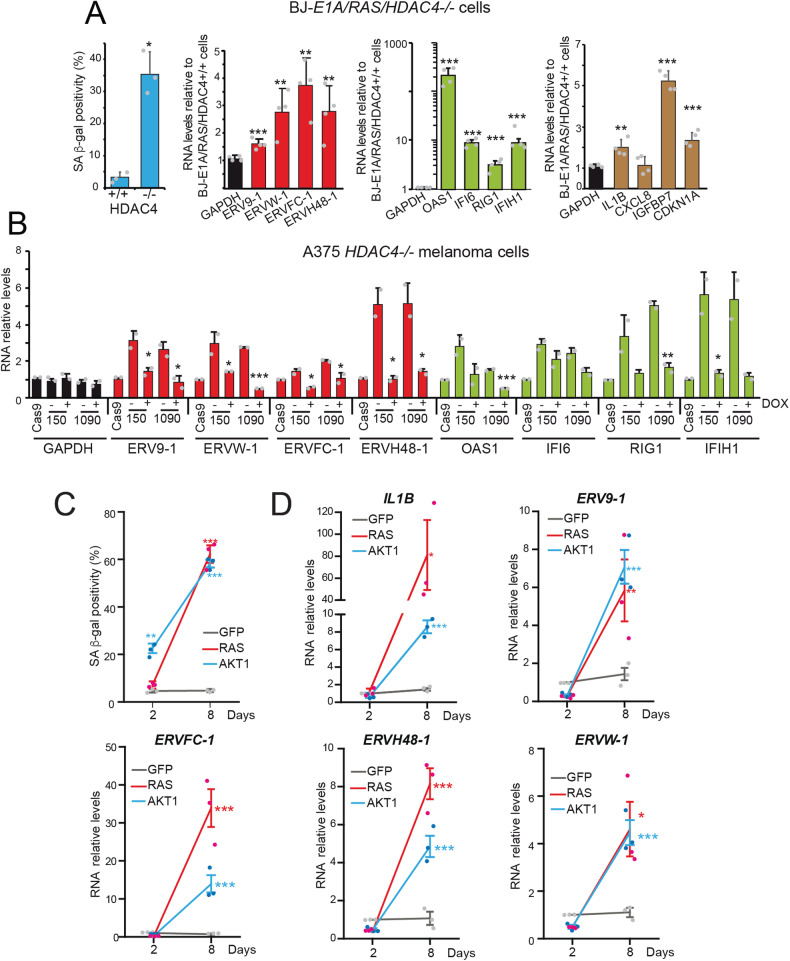
Upregulation of ERVs and of the IFN response in different models of OIS. **A** (left) SA β-gal positivity in BJ-*hTERT/E1A/RAS* WT or knocked out for HDAC4; (right) Expression levels of the indicated ERVs, ISGs and SASP genes in BJ-*hTERT/E1A/RAS* knocked out for *HDAC4*. RNAs were extracted and processed for qRT-PCR. Data are relative BJ-*hTERT/E1A/RAS* expressing HDAC4 (set to 1). Mean ± SD; *n* = 3. *p < 0.05, **p < 0.01, ***p < 0.001 *t*-test. **B** Expression levels of the indicated ERVs, ISGs and SASP genes in A375 melanoma cells knocked out for HDAC4. RNAs were extracted and processed for qRT-PCR. Data are relative to wt A375 cells. Mean ± SD; *n* = 2 for each clone. *p < 0.05, **p < 0.01, ***p < 0.001 paired *t*-test between plus and minus DOX conditions. **C** SA β-gal positivity in BJ-*hTERT* cells expressing inducible versions of AKT or RAS. Cells grown for the indicated days in the presence of 4OHT were fixed, processed for SA-β-gal assay and scored at the microscope. *n* = 3, **p < 0.01, ***p < 0.001, Dunn’s Multiple Comparison Test. **D** Expression levels of the indicated ERVs and of *IL1B* in BJ-*hTERT/GFP*, BJ-*hTERT/AKT* and BJ-*hTERT/RAS* grown for the indicated days in the presence of 4OHT. RNAs were extracted and processed for qRT-PCR. Data are relative to BJ-*hTERT/GFP* without 4OHT. Mean ± SD; *n* = 3. *p < 0.05, **p < 0.01, ***p < 0.001, Dunn’s Multiple Comparison Test.

To further confirm that HDAC4 is associated with upregulation of ERVs during senescence, we chose a classical model of OIS: BJ human diploid fibroblasts co-expressing RAS (G12V). In these cells, RAS triggers senescence and co-expression of the viral oncoprotein E1A stop the program. BJ-*RAS/E1A* cells, after elimination of HDAC4 by CRISPR/Cas9, underwent senescence. This model was previously fully characterized [8]. Here we quantified the expression levels of a group of ERVs. Entry into senescence is associated with upregulation of all ERVs tested, including *ERVW-1*, which was not upregulated in LMS cells, and IFN response genes (Fig. 2A). We confirmed the upregulation of ERVs in another model of HDAC4-dependent senescence to exclude lineage-specific effects. In A375 melanoma cells silenced for HDAC4, we expressed a Dox-inducible version of HDAC4. In this way, the cell line can be propagated in culture. After removal of Dox, cells enter senescence (Fig. S1B). Also in this model, senescence is coupled to upregulation of the selected ERVs and genes of the IFN response (Fig. 2B).

Finally, we investigated whether these ERVs are also upregulated in additional models of OIS triggered by strong oncogenes. We used BJ-*hTERT* cells expressing inducible versions of HRAS-G12V or myr-AKT1 (Fig S1C). Following oncogene induction, after an initial proliferative burst, these cells enter senescence 8 days later. SA-β-gal assay (Fig. 2C), *IL1B* upregulation (SASP gene) (Fig. 2D) and the upregulation of CDKN1A (Fig. S1C) confirmed the induction of OIS. Also in this model, all ERVs tested were upregulated (Fig. 2D). Altogether, these results demonstrate that *ERV9-1*, *ERVW-1*, *ERVFC1-1* and *ERVH48-1* are part of a common genetic program that is engaged in different forms of senescence.

Interestingly, in MCF10A cells, knockout of HDAC7, the most abundantly expressed class IIa HDAC, induces expression of *ERV9-1* and *ERVH48-1* but not *ERVW-1* and *ERVFC1-1*. These cells do not enter senescence in response to the deletion of HDAC7 [31], possibly because of the deletion within the *CDKN2A* locus [32]. Interestingly, RAS also triggered the upregulation of *ERVFC1-1*, and the concomitant deletion of HDAC7 further enhanced the upregulation of these three ERVs and the IFN response (Fig. S2).

Therefore, the link to ERVs is not limited to HDAC4 but also involves other class IIa HDACs as general regulators to achieve directly or indirectly the silencing of defined ERVs.

### Deletion of HDAC4 leads to a decrease in H3K27me3 at several intergenic ERVs

**Fig. 3:**
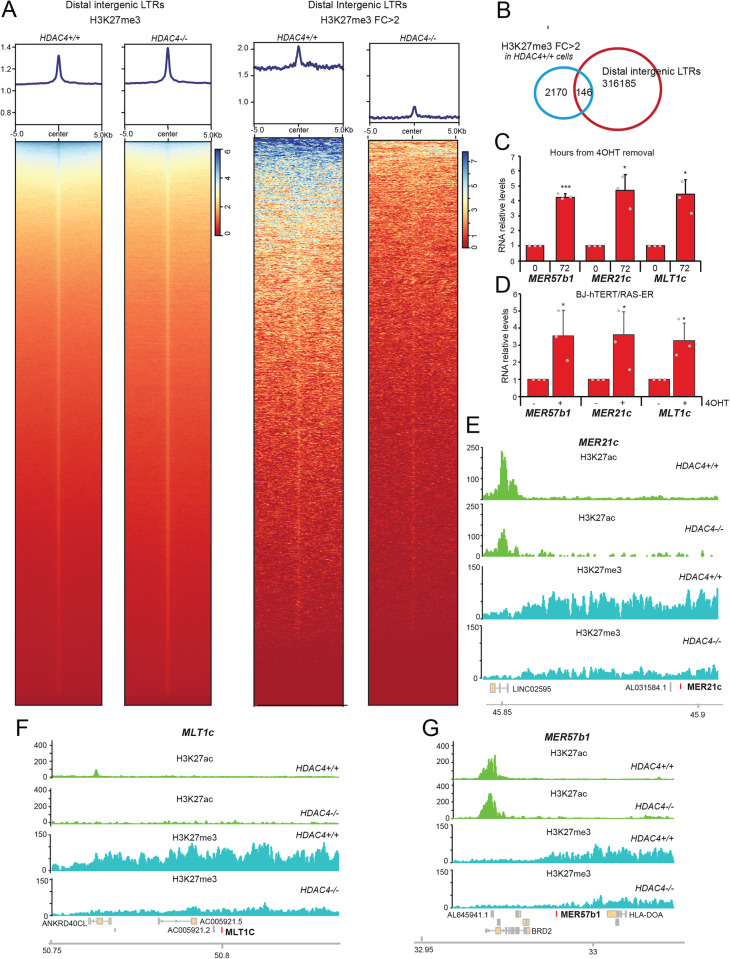
H3K27 demethylation at intergenic LTRs. **A** Heat-maps of the H3K27me3 signal distribution in SK-LMS-1^*HDAC4-/-/HDAC4PAM-ER*^ WT or KO for HDAC4. (left) a region of ±5 kb around intergenic LTRs and (right) a region of ±5 kb around the intergenic LTRs which show a reduction of at least 2 folds in H3K27me3 signal after HDAC4 knock-out. **B** Venn diagram showing the number of intergenic LTRs where H3K27me3 is reduced by at least 2 folds after the knockout of HDAC4. **C** Expression levels of the indicated ERVs in SK-LMS-1^*HDAC4-/-/HDAC4PAM-ER*^ grown in the presence or absence of 4OHT. Data are relative to the presence of 4OHT. Mean ± SD; *n* = 3. *p < 0.05, **p < 0.01, ***p < 0.001, paired t-test between plus and minus 4OHT conditions. **D** Expression levels of the indicated ERVs in BJ-*hTERT/RAS-ER* grown in the presence or absence of 4OHT. Data are relative to the absence of 4OHT. Mean ± SD; *n* = 3. *p < 0.05, **p < 0.01, ***p < 0.001, paired *t*-test between plus and minus 4OHT conditions. **E**–**G** Detailed view of H3K27me3 and H3K27ac tracks at three representatives intergenic ERVs (*MER21c, MLT1c*, and *MER57b1*), upregulated after HDAC4 knock-out and showing a reduction of the H3K27me3 signal. Gene structure and chromosomal location are shown.

In cells, ERVs are often silenced to prevent their abnormal transcription. Epigenetic markers for silenced chromatin such as H3K9me3, H3K27me3, and DNA methylation are deposited at ERVs loci [33–35]. To better understand the mechanisms that trigger the expression of ERVs during OIS, we examined genome-wide variations in H3K27me3 distribution in SK-LMS-1^*HDAC4-/-/HDAC4PAM-ER*^ cells undergoing senescence. ChIP-seq analysis revealed that deletion of HDAC4 does not cause overt changes in H3K27me3 (Fig. 3A) [8]. Instead, a selected number of genomic regions show a consistent reduction in H3K27me3, as shown by the heat-map (H3K27me3 signal >2 folds). To understand whether these regions contained ERVs, they were crossed with distal intergenic LTRs (*n* = 316.185), which revealed 146 regions containing ERVs whose H3K27me3 levels were reduced after HDAC4 KO (Fig. 3B and Table S2). Importantly, significant H3K27me3 demethylation was observed after HDAC4 depletion in the genomic loci of *ERV9-1* and *ERVFC1-1*, but not in the ORF containing *ERVW-1* and *ERVH48-1* (Table S2).

The up-regulation of the transcripts of three of these ERVs (*MER57B1*, *MER21C* and *MLT1C*) was validated by qRT-PCR (Fig. 3C). Moreover, their upregulation also occurred in BJ-*hTERT* fibroblasts during RAS-induced OIS (Fig. 3D).

Accordingly, genomic loci containing *MER57B1*, *MER21C* and *MLT1C* show a dramatic reduction of H3K27me3 signals after HDAC4 depletion, whereas H3K27ac levels remain largely unchanged (Fig. 3E–G). Importantly, upregulation of ERVs during senescence may also involve additional epigenetic mechanisms. For example, the *METTL9* locus is characterized by an increase in H3K27ac during senescence triggered by HDAC4 depletion (Fig. S3).

In conclusion, these results confirm that the upregulation of ERVs during senescence is a general and conserved phenomenon involving epigenetic resetting.

### *MER57B1, MER21C, ERV9-1* and *ERVFC-1* are not upregulated in response to DNA damage

**Fig. 4:**
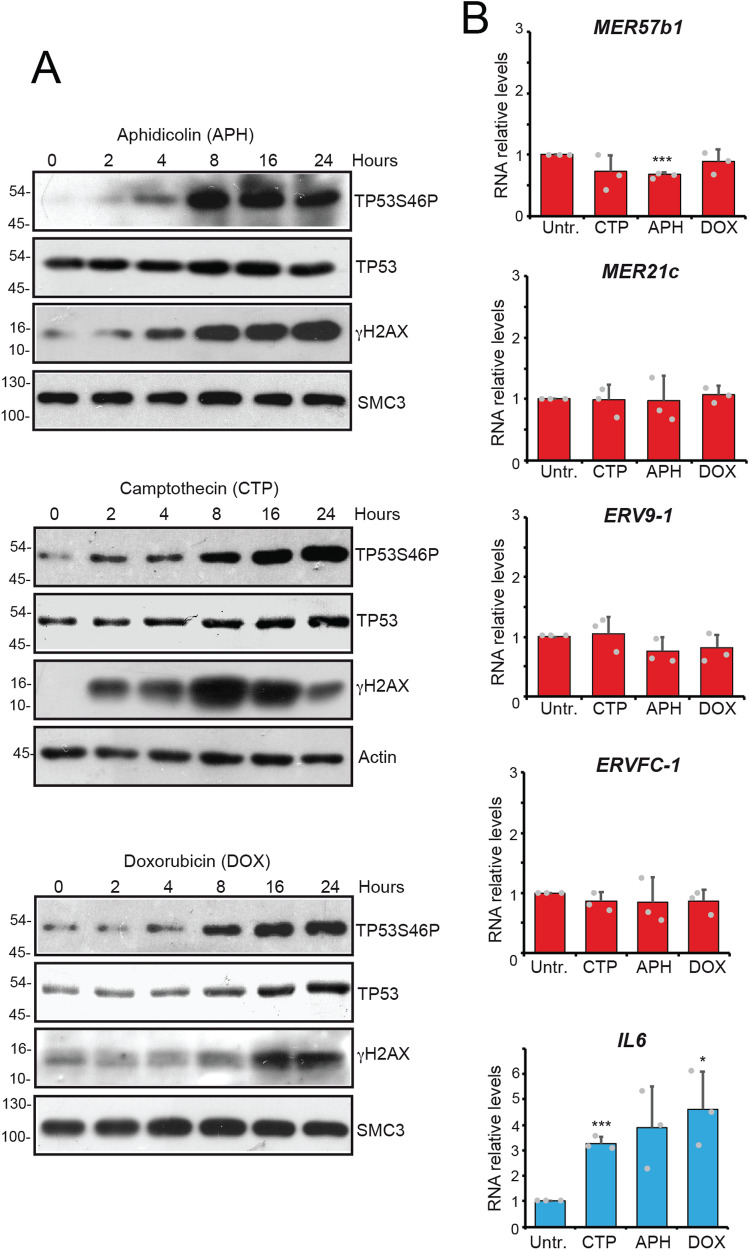
The DNA damage response does not upregulate the ERVs of senescence. **A** Immunoblot analysis of TP53 activation and DNA damage (γH2AX). Lysates obtained from SK-LMS-1 cells, treated for the indicated times with 6.25 μM Camptothecin (CPT), 1 μM Aphidicolin (APH) or 100 nM Doxorubicin (DOX), were immunoblotted using the indicated antibodies. Actin and SMC3 were used as loading control. **B** Expression levels of the indicated ERVs in SK-LMS-1 treated for 24 h with 6.25 μM CPT, 1 μM APH or 100 nM DOX. Data are relative to untreated cells. Mean ± SD; *n* = 3. *p < 0.05, **p < 0.01, ***p < 0.001, Dunn’s Multiple Comparison Test.

It has been reported that induction of ERVs is associated with the presence of DNA damage and that senescence is associated with the accumulation of DNA damage [36, 37]. Similarly, the activation of IFN response was previously correlated to the activation of cGAS/STING pathway as a consequence of the accumulation of cytoplasmic DNA scars [1, 2, 6]. To clarify the correlation between ERVs upregulated in senescent cells and the DNA damage response (DDR), SK-LMS-1 cells were treated with various DNA-damaging agents: aphidicolin (APH), camptothecin (CPT) and doxorubicin (DOX). All compounds induced DNA damage (γH2AX positivity) and activation of the DDR, which was confirmed by TP53 phosphorylation and stabilization (Fig. 4A). The expression of the investigated ERVs was analyzed within 24 h after treatment to exclude their modulation as a secondary event after the activation of senescence by chemotherapeutic agents [37]. Their expression remained unchanged during DDR (Fig. 4B). Therefore, we can affirm that upregulation of these ERVs is specifically correlated with senescence.

### dsRNAs and the regulation of senescence

Transcription by LTRs can generate dsRNAs that are recognized by selected receptors in the cytoplasm, endosomal compartment, or cell surface. These receptors activate signaling pathways that induce type I and type III interferon (IFN) expression and increased immunogenicity [38, 39]. TLR3 is the major receptor for dsRNAs at the cell surface, whereas RIG1 (retinoic acid-inducible gene I) and IFIH1/MDA5 (interferon induced with helicase C domain 1/melanoma differentiation-associated gene 5) are the major cytosolic receptors. RIG1 mainly recognizes 5’-triphosphate sRNAs and short dsRNA (<1 kb). IFIH1, on the other hand, preferentially binds long dsRNAs (>2 kb) [40]. Engagement of these receptors leads to activation of MAVS (mitochondrial antiviral signaling) [41].

Several studies have addressed the contribution of IFN response in senescence and aging [42]. The upregulation of ERVs during senescence may be responsible for the onset of the IFN response, thus contributing to senescence.

**Fig. 5:**
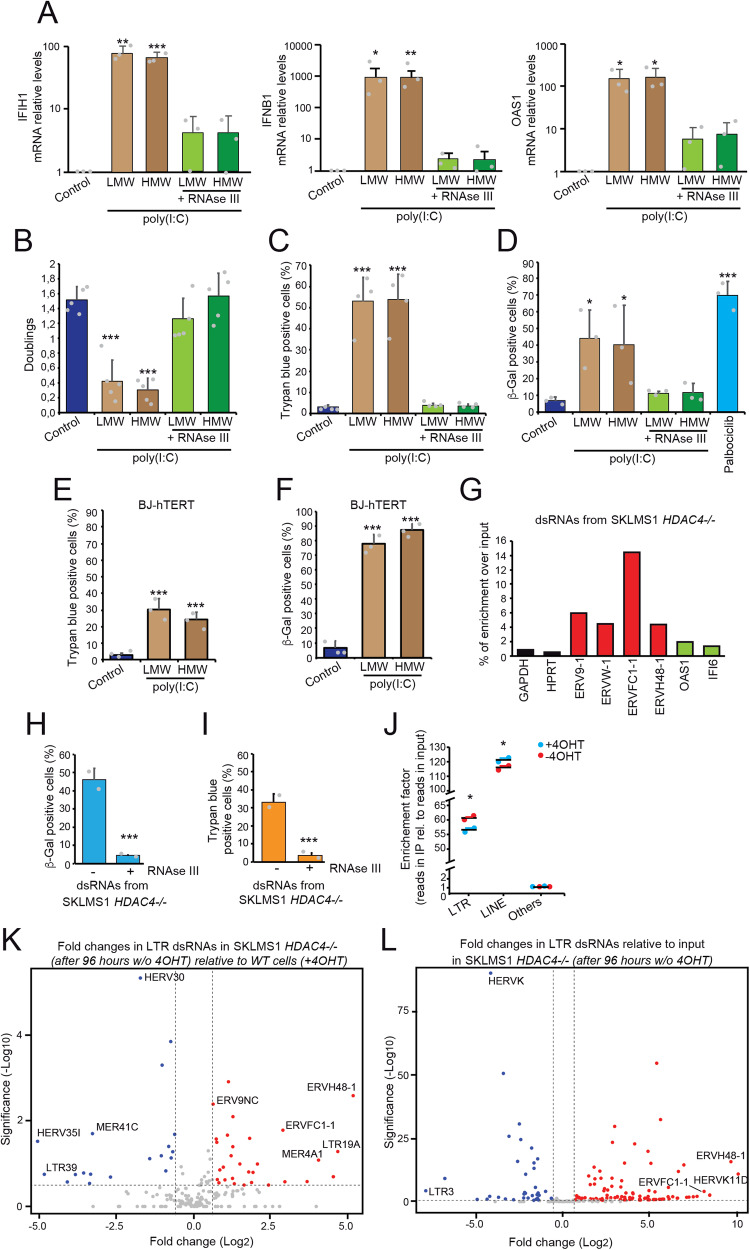
dsRNAs can trigger cell death and senescence. **A** Expression levels of the indicated ISGs. SK-LMS-1 cells were transfected for 48 h with 10 ng/ml of low molecular (LMW) and high molecular (HMW) weight poly (I:C), pre-digested or not for 30 min with 50U of RNAse III. Data are relative to untreated cells. Mean ± SD; *n* = 3. *p < 0.05, **p < 0.01, ***p < 0.001, paired *t*-test relative to control condition. **B** SK-LMS-1 cells were treated as in (**A**) and the proliferative potential was evaluated as doubling. Mean ± SD; *n* = 5. *p < 0.05, **p < 0.01, ***p < 0.001, paired *t*-test relative to control condition. **C** SK-LMS-1 cells were treated as in (**A**) and cell death was evaluated by Trypan blue staining. Mean ± SD; *n* = 4. *p < 0.05, **p < 0.01, ***p < 0.001, paired *t*-test relative to control condition. **D** SK-LMS-1 cells were treated as in (**A**) and senescence was evaluated by SA β-gal staining. Palbociclib was used 3 µM for 48 h. Mean ± SD; *n* = 3. *p < 0.05, **p < 0.01, ***p < 0.001, paired *t*-test relative to control condition. **E** BJ-*hTERT* cells were treated as in (**A**) and cell death was evaluated by Trypan blue staining. Mean ± SD; *n* = 3. *p < 0.05, **p < 0.01, ***p < 0.001, paired *t*-test relative to control condition. **F** BJ-*hTERT* cells were treated as in (**A**) and senescence was evaluated after 11 days from transfection by SA β-gal staining. Mean ± SD; *n* = 3. *p < 0.05, **p < 0.01, ***p < 0.001, paired *t*-test relative to control condition. **G** Quantification of the enrichment over input of the indicated species of RNAs in the preparation of double-stranded enriched RNAs (dsRNAs) from SK-LMS-1^*HDAC4-/-/HDAC4PAM-ER*^ grown in the absence of 4OHT for 4 days. The J2 antibody was used for the purification of dsRNAs. **H** Analysis of SA β-gal positivity in SK-LMS-1 cells transfected for 72 h with 30 pmoles of dsRNAs obtained as described in G), and pre-digested or not for 30’ with 50U of RNAse III. Mean ± SD; *n* = 3. *p < 0.05, **p < 0.01, ***p < 0.001, *t*-test. **I** Cell death as determined by Trypan blue assay positivity in SK-LMS-1 cells transfected for 72 h with 30 pmoles of dsRNAs obtained as described in (**G**), and pre-digested or not for 30’ with 50U of RNAse III. Mean ± SD; *n* = 3. *p < 0.05, **p < 0.01, ***p < 0.001, *t*-test. **J** Enrichment (expressed as multiplication factor of total read counts) of the indicated non-coding elements in dsRNA-seq samples obtained from SK-LMS-1^*HDAC4-/-/HDAC4PAM-ER*^ grown in the presence or absence of 4OHT for 4 days, as indicated. *p < 0.05, Mann–Whitney. **K** Volcano plot that shows statistical significance (-log10(*P* value)) versus magnitude of change (log2(fold change)) of the abundance of ERVs in dsRNA-seq samples obtained from SK-LMS-1^*HDAC4-/-/HDAC4PAM-ER*^ grown in the absence of 4OHT for 4 days, in respect to cells grown in the presence of 4OHT. **L** Volcano plot that shows statistical significance (-log10(*P* value)) versus magnitude of change (log2(fold change)) of the abundance of ERVs in dsRNA-seq samples obtained from SK-LMS-1^*HDAC4-/-/HDAC4PAM-ER*^ grown in the absence of 4OHT for 4 days, in respect to the total RNA extracted from cells grown in the presence of 4OHT.

First, we transfected the synthetic poly(I:C) (polyriboinosinic:polyribocytidylic acid), as a mimic of the dsRNA generated by ERVs transcription. In SK-LMS-1 cells, poly(I:C)-HMW or poly(I:C)-LMW trigger dramatic upregulation of IFN genes *IFIH1, IFNB1* and *OAS1*. Pretreatment with RNAse III impaired the upregulation of IFN response, proving the specificity. No differences were detectable between LMW and HMW poly(I:C), suggesting the integrity of both IFIH1 and RIG1 signaling (Fig. 5A). We next examined proliferative potential (Fig. 5B), induction of cell death (Fig. 5C) and accumulation of senescent cells (Fig. 5D). Poly(I:C) transfections lead to cell cycle arrest, cell death, but also accumulation of SA β-gal positive cells. The CDK4 inhibitor palbociclib was used as a positive control for senescence. Induction of cell death (Fig. 5E) and of senescence (Fig. 5F) in response to transfection of LMW and HMW poly(I:C), was confirmed in immortalized human fibroblasts (BJ-*hTERT*).

Finally, dsRNAs were immunopurified from senescent SK-LMS -1^*HDAC4-/-/HDAC4PAM- ER*^ cells grown in the absence of HDAC4 (-4OHT) using the J2 antibody. The enrichment of ERVs is shown in Fig. 5G. Transfection of cellular dsRNAs generated during senescence was sufficient to induce senescence (Fig. 5H). Of note, a proportion of cells that received dsRNAs isolated from senescent cells activated an apoptotic program (Fig. 5I). Treatment with RNAse III confirmed the specificity of dsRNA activity.

To characterize the dsRNAs present in senescent cells, we performed RIP-seq using the J2 antibody in proliferating SK-LMS -1^*HDAC4-/-/HDAC4PAM- ER*^ cells and in cells entering senescence after the depletion of HDAC4 (96 h -4OHT). PCA (principal component analysis) confirmed the high enrichment of specific dsRNA species compared to the input during immunoprecipitation and the good reproducibility of the results (Fig. S4A). By restricting the analysis to ERVs alone, the distinction between cells undergoing senescence and proliferating cells becomes even clearer, suggesting that the differential expression of ERVs and their folding to dsRNA are meaningful parameters to distinguish the two states (Fig. S4B). Consistently with previous data [43], retrotransposons are the most enriched elements in dsRNA preparations purified from cells, whether proliferating or senescent (Fig. 5J). ERVs are the second most enriched class of elements in dsRNA purifications but, importantly, the only class that is significantly enriched in dsRNA species isolated from senescent cells (Fig. 5J). Specifically, 32 ERV entities were identified that are selectively enriched in dsRNA preparations from senescent cells compared to proliferating cells (Fig. 5K and Table S3). Of the ERVs examined throughout the manuscript (*ERV9-1*, *ERVW-1*, *ERVFC1-1*, *ERVH48-1*), *ERVFC1-1* and *ERVH48-1* were among the most upregulated ERVs folded as dsRNA in SK-LMS-1 senescent cells, whereas *ERVW-1* was not upregulated in this model of senescence, as expected (Fig. 1J).

*ERV9-1* was not found to be significantly enriched as dsRNA during senescence. However, when the upregulation of *ERV9-1* family entities was analyzed, upregulation of most of its entities was observed, although not significantly in virtue of the scattering of data (Fig. S5). *ERVFC1-1* and *ERVH48-1* are also among the ERVs that most efficiently produce dsRNAs (Fig. 5L and Table S4). By contrast *HERVK* [44] was the most significant downregulated dsRNAs ERV under senescence induced after HDAC4 KO.

Finally, we identified a core signature of 42 ERVs (Fig. S4C and Table 1) that are significantly enriched upon dsRNAs purification and are highly induced in cells that become senescent after HDAC4 depletion compared to proliferating cells. 87% of dysregulated ERVs belong to class I ERVs and 57.5% to the *HUERSP*, *HEPSI* and *HERVHF* supergroups (Fig. S4D) [45].

**Table 1:** 42 ERV elements significantly enriched ( | log2FC | >1, FDR < 0.30) in dsRNA-seq compared to input (total RNA) and in dsRNA-seq preparations from HDAC4-depleted SK-LMS-1 cells undergoing senescence compared to proliferating HDAC4-expressing cells.

id	log2_FC (−4OHT vs +4OHT)	log2_FC (dsRNA KO vs input WT
HERVH48#LTR/ERV1	5.134720392	9.610618271
LTR19A#LTR/ERV1	4.638675483	3.25809821
MER4A1_v#LTR/ERV1	4.017911632	4.807503556
LTR28#LTR/ERV1	2.932275279	4.369902777
HERV-Fc1_LTR1_LTR3#LTR/ERV1	2.868725565	8.068409738
LTR1B#LTR/ERV1	2.764851952	2.764851952
LTR36#LTR/ERV1	2.764851952	2.764851952
LTR9#LTR/ERV1	2.758767057	6.055371874
L1M4_orf2#LINE/L1	2.553590805	3.990671258
HERVIP10B3#LTR/ERV1	2.297203241	5.59320468
LOR1b#LTR/ERV1	2.102801706	1.820381777
HUERS-P3#LTR/ERV1	2.046840768	2.480967407
LTR8A#LTR/ERV1	2.039271371	3.476930306
LTR8B#LTR/ERV1	1.999548071	5.296157619
Arthur2#DNA/hAT-Tip100	1.961591741	1.961591741
LTR24B#LTR/ERV1	1.961591741	1.961591741
LTR49-int#LTR/ERV1	1.961591741	1.961591741
LTR57-int#LTR/ERVL	1.961591741	1.961591741
MER31-int#LTR/ERV1	1.961591741	1.961591741
MER65-int#LTR/ERV1	1.961591741	1.961591741
MER72#LTR/ERV1	1.961591741	1.961591741
MER83B#LTR/ERV1	1.961591741	1.961591741
LTR54#LTR/ERV1	1.958120775	1.958120775
MER34B#LTR/ERV1	1.958120775	1.958120775
MER41D#LTR/ERV1	1.958120775	1.958120775
MER57B1#LTR/ERV1	1.958120775	1.958120775
MER61C#LTR/ERV1	1.958120775	1.958120775
MLT2B2#LTR/ERVL	1.958120775	1.958120775
Tigger5#DNA/TcMar-Tigger	1.873695804	4.028334124
HERVFH19#LTR/ERV1	1.765867216	6.592533106
L1MCa_5end#LINE/L1	1.707430414	7.483920385
LTR18B#LTR/ERVL	1.624110228	4.92010988
LTR61#LTR/ERV1	1.605180488	2.311061997
LTR6A#LTR/ERV1	1.481433701	1.54289511
MER75B#DNA/PiggyBac	1.457641417	5.940566451
L1PA17_3end#LINE/L1	1.262693279	1.662696658
LTR6B#LTR/ERV1	1.257530299	2.77467016
MER83#LTR/ERV1	1.243166352	2.673094326
MER51A#LTR/ERV1	1.156752606	7.663355535
LTR9D#LTR/ERV1	1.116852126	4.412851778
MER30#DNA/hAT-Charlie	1.07807492	4.831540176
MER57A1#LTR/ERV1	1.028041275	2.948085407

Fold induction represents the average of the aggregate of all entities forming the subfamily represented by the indicated ERV.

Overall, our analysis highlights how certain supergroups of ERVs are overrepresented in senescent cells and folded as dsRNAs and therefore represent possible ligands for intracellular receptors of dsRNA species.

### dsRNAs receptors and the regulation of senescence

**Fig. 6:**
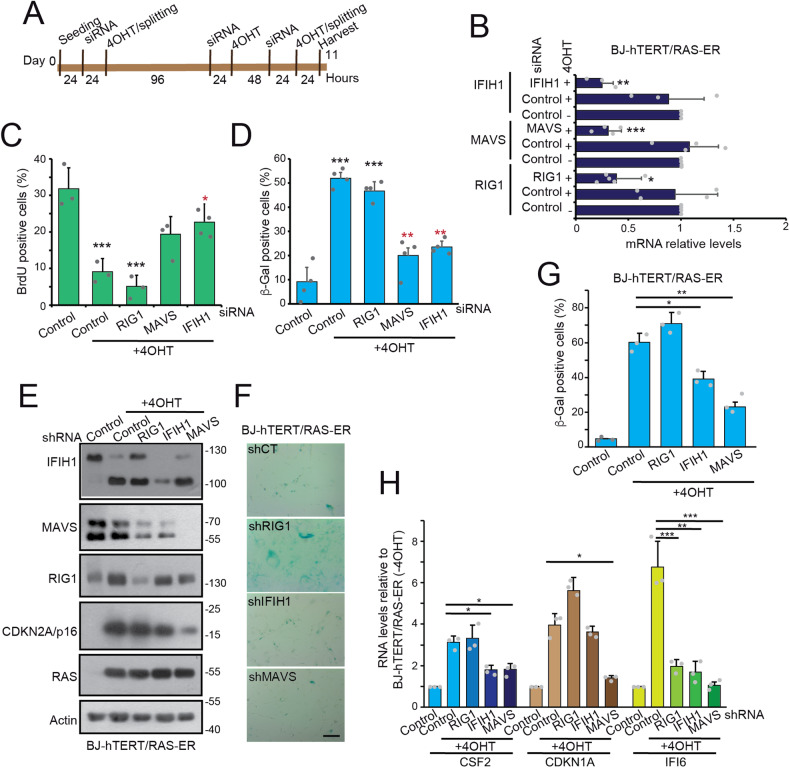
dsRNAs receptors and the regulation of senescence. **A** Schematic representation of the experimental protocol used to evaluate the effect of gene silencing during OIS in BJ-*hTERT/RAS-ER* cells. **B** Expression levels of the indicated genes in cells silenced with the relative siRNA as schematized in (**A**). Mean ± SD; *n* = 3. *p < 0.05, **p < 0.01, ***p < 0.001, *t*-test. **C** Analysis of BrdU incorporation in BJ-*hTERT/RAS-ER* cells, treated as schematized in (**A**). Significance is relative to cells grown without 4OHT and transfected with control siRNA. Mean ± SD; *n* = 4. *p < 0.05, **p < 0.01, ***p < 0.001, *t*-test relative to control. **D** SA β-gal positivity in BJ-*hTERT/RAS-ER* cells, treated as schematized in (**A**). Significance is relative to cells grown without 4OHT and transfected with control siRNA. Mean ± SD; *n* = 4. *p < 0.05, **p < 0.01, ***p < 0.001, *t*-test relative to control. **E** Immunoblot analysis in BJ-*hTERT/RAS-ER* cells silenced for RIG1, IFIH1 and MAVS. Lysates obtained from cells grown or not in the presence of 4OHT were immunoblotted and probed with the indicated antibodies. **F** SA β-gal positivity in BJ-*hTERT/RAS-ER* cells silenced for RIG1, IFIH1 and MAVS, after treatment with 4OHT. Bar = 50 µm. **G** Quantitative analysis of SA-β-gal positivity BJ-*hTERT/RAS-ER* cells knocked-down for RIG1, IFIH1 and MAVS grown in the presence or not of 4OHT. Mean ± SD; *n* = 3. *p < 0.05, **p < 0.01, ***p < 0.001, *t*-test relative to shRNA control. **H** Expression levels of the indicated genes in BJ-*hTERT/RAS-ER* cells silenced for RIG1, IFIH1 and MAVS, grown in the presence or not of 4OHT. RNAs were extracted and processed for qRT-PCR. Data are relative to BJ-*hTERT/RAS-ER* control cells grown in the absence of 4OHT. Mean ± SD; *n* = 3. *p < 0.05, **p < 0.01, ***p < 0.001, *t*-test.

To understand the contribution of the dsRNA signaling pathway to the induction of senescence, the expression of the components RIG1, IFIH1 and MAVS was silenced in BJ-*hTERT* cells during RAS-induced senescence. We optimized a specific protocol to maintain silencing of the genes throughout the experiments (Fig. 6A). RT-PCR experiments confirmed the efficiency of silencing (Fig. 6B). The activities of IFIH1 and MAVS, but not RIG1, contribute to cell cycle arrest (Fig. 6C) and the appearance of senescence (Fig. 6D).

To confirm these results, we stably knocked-down the expression of RIG1, IFIH1 and MAVS with shRNAs by lentiviral infection (Fig. 6E). RAS-induced OIS in BJ-*hTER*T cells is characterized by the appearance of a rapidly migrating form of IFIH1. Importantly, upregulation of p16/CDKN2A in response to RAS was dramatically downregulated in MAVS-silenced cells. When IFIH1 was downregulated p16/CDKN2A was modestly repressed. RIG1 silencing has no effect on the upregulation of p16/CDKN2A during RAS-induced senescence. SA β−gal staining confirmed the role of MAVS and IFIH1 in the regulation of OIS and excluded a contribution of RIG1 (Fig. 6F, G). Expression of *CSF2*, *CDKN1A* and *IF16* was examined to monitor SASP, cell cycle arrest and activation of IFN response. Expression of MAVS and IFIH1 was required for maintenance of *CSF2* expression. *CDKN1A* upregulation was specifically dependent on MAVS, while *IF16* upregulation in response to OIS was affected by all sensors (Fig. 6H).

### A MAVS dependent pathway sustains senescence

MAVS emerged as an important player of the RAS-induced senescence. To further confirm the role of MAVS, we again used the HDAC4-dependent model of senescence. SK-LMS-1^*HDAC4-/-/HDAC4PAM-ER*^ cells were engineered to express the dominant negative (DN) version of MAVS (MAVSΔCARD) [46]. As positive control, we used a mutant version of TP53 (TP53^R175H^) which is known to escape OIS [47]. Since the role of TP53 in OIS is well established, we used only one clone.

**Fig. 7:**
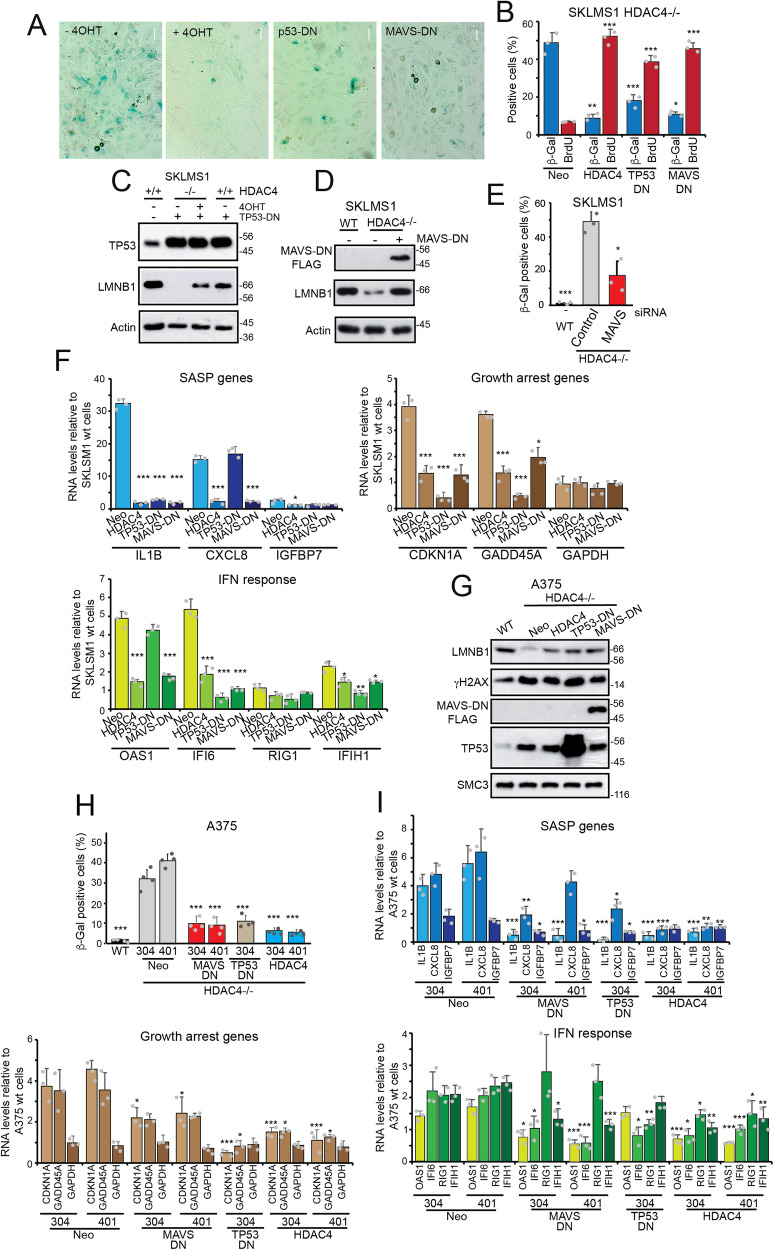
MAVS and the regulation of senescence. **A** Representative microscopic images of SA β-gal stained SK-LMS-1^*HDAC4-/-/HDAC4PAM*^ grown in the presence or not of 4OHT and expressing the indicated transgenes (scale bar 40 μm). **B** Quantitative analysis of SA β-gal positivity and BrdU incorporation in SK-LMS-1^*HDAC4-/-/HDAC4*^ grown in the presence or not of 4OHT and expressing the indicated transgenes. Mean ± SD; *n* = 3. *p < 0.05, **p < 0.01, ***p < 0.001, paired *t*-test relative to NEO condition. **C** Immunoblot analysis using the indicated antibodies. Whole cell extracts were obtained from SK-LMS-1^*HDAC4-/-/HDAC4PAM-ER*^ grown in the presence or not of 4OHT and expressing or not TP53. **D** Immunoblot analysis using the indicated antibodies. Whole cell extracts were obtained from SK-LMS-1^*HDAC4-/-/HDAC4PAM-ER*^ grown in the presence or not of 4OHT and expressing or not MAVS-DN. **E** Quantitative analysis of SA β-gal positivity in SK-LMS-1^*HDAC4-/-/HDAC4PAM-ER*^ grown in the presence or not of 4OHT and transfected with the indicated siRNAs. Mean ± SD; *n* = 3. **p < 0.01, paired t-test relative to *HDAC4*^*−/−*^ control condition. **F** Expression levels of the indicated genes in SK-LMS-1^*HDAC4-/-/HDAC4PAM-ER*^ knocked out for *HDAC4* and re-expressing HDAC4 or the indicated transgenes. RNAs were extracted and processed for qRT-PCR. Data are relative to SK-LMS-1^*HDAC4-/-/HDAC4PAM-ER*^ grown in the absence of 4OHT. Mean ± SD; *n* = 3. *p < 0.05, **p < 0.01, ***p < 0.001, paired *t*-test relative to NEO condition. **G** Immunoblot analysis using the indicated antibodies. Whole cell extracts were obtained from A375 cells grown WT or KO for HDAC4 and expressing the indicated transgenes. HDAC4 expression was maintained by adding Dox to the culture medium. **H** Quantitative analysis of SA β-gal positivity in A375 cells WT or KO for HDAC4 and expressing the indicated transgenes. HDAC4 expression was maintained by adding Dox to the culture medium. Mean ± SD; *n* = 4. *p < 0.05, **p < 0.01, ***p < 0.001, paired *t*-test relative to NEO condition. **I** Expression levels of the indicated genes in A375 cells WT or KO for HDAC4 and expressing the indicated transgenes. HDAC4 expression was maintained by adding Dox to the culture medium. RNAs were extracted and processed for qRT-PCR. Data are relative to SK-LMS-1^*HDAC4-/-/HDAC4PAM-ER*^ grown in the absence of 4OHT. Mean ± SD; *n* = 3. *p < 0.05, **p < 0.01, ***p < 0.001, paired *t*-test relative to NEO condition.

Expression of TP53-DN and of MAVS-DN in *HDAC4*^*−/−*^ cells reduced SA-β-gal positivity and rescued proliferation, similar to the reintroduction of HDAC4 (Fig. 7A, B). The anti-senescence effect was confirmed by the rescue of LMNB1 expression (Fig. 7C, D). To confirm the involvement of MAVS in senescence, we also silenced its expression in SK-LMS-1^*HDAC4−/−*^ cells (Fig. 7E). Interfering with MAVS suppressed activation of the senescence program in terms of SASP, cell cycle genes and IFN response, similar to re-expression of HDAC4. We observed that TP53 had a much stronger effect on regulating growth arrest genes compared with HDAC4 and MAVS. In contrast, upregulation of *CXCL8* is highly dependent on the activities of HDAC4 and MAVS (Fig. 7F).

To further confirm the contribution of MAVS to senescence, A375 melanoma cells in which *HDAC4* was knocked-out, were engineered to express MAVS-DN. The Dox-inducible system was used to re-express HDAC4, and two different clones of knocked-out cells were selected. We also overexpressed TP53-DN as a positive control (Fig. 7G). All different transgenes counteracted LMNB1 downregulation and senescence induced by *HDAC4* knock-out (Fig. 7G, H).

Expression of SASP genes *IL1B* and *CXCL8* was efficiently repressed by HDAC4, whereas TP53-DN and MAVS-DN repressed *CXCL8* less efficiently (Fig. 7I). TP53-DN strongly suppressed the upregulation of cell cycle genes *CDKN1A* and *GADD45A* compared with MAVS-DN. Instead, MAVS-DN repressed IFN response genes more efficiently, apart from *RIG1* (Fig. 7I).

In summary, ERVs that are upregulated during senescence contribute to the accumulation of dsRNAs that can activate IFIH1-MAVS signaling and the antiviral response, thus contributing to the maintenance of the senescence program.

## Discussion

In this manuscript, we provide evidence for upregulation of ERV transcripts in different models of cellular senescence. The ERVs here investigated were found upregulated during replicative senescence, OIS, and during HDAC4 deletion-induced senescence. However, these ERVs are not upregulated in response to genotoxic stress. This finding suggests that these ERVs are part of a central genetic program that is universally deployed in various forms of senescence in human cells.

Induction of ERVs during senescence is the result of profound epigenomic rearrangements. Global genomic analysis revealed that a selected number of intergenic LTRs exhibit a marked reduction in H3K27me3 during senescence. However, it is plausible that other epigenetic mechanisms, involved in the abrogation of ERV expression [48, 49], are discharged during senescence. Accordingly, senescence and aging are known to be characterized by methylation changes that can subvert the transcriptional landscape [50].

The upregulation of retroviral elements during senescence and aging has been reported previously [23, 24]. *HERV1* and *L1* and, to a lesser extent, *HERVK* and *HERVL* are deregulated during replicative senescence and in fibroblasts with Li-Fraumeni syndrome in which senescence was induced by genotoxic and oxidative drugs [23]. Similar to our observation, this upregulation is the result of a change in the heterochromatin/euchromatin compartments [23]. Recently, it has been reported that integrated human *ERVK* (*HML-2*) is activated by epigenetic resetting during senescence and can assemble into functional virions that can maintain the senescence program. *HERVK*, once secreted, can trigger senescence in neighboring cell [44]. In our dsRNA purification from senescent cells, HERVK is the most highly depopulated ERV. Therefore, our data suggest that HERVK is not only strongly induced but also strongly processed in senescent cells, resulting in minimal accumulation of dsRNAs.

The expression of ERVs can support a pro-inflammatory microenvironment through the expression of ISGs [26–28]. Accordingly, we found that ERVs upregulation in senescence is closely associated with activation of the IFN response in all models of cellular senescence examined.

Indeed, bidirectional transcription of LTRs or self-pairing between tandem ERV genome copies promotes folding of ERV transcripts into dsRNA structures [51]. Consistent with this, we observed that dsRNAs accumulate in the cytoplasm of senescent cells. These dsRNA species are mainly composed by LINE and ERVs elements and they could be recognized by RLRs family members RIG1 and IFIH1. These receptors can trigger a strong inflammatory-immune response through the engagement of MAVS [39].

Exogenous administration of dsRNA species derived from a cellular model of HDAC4-dependent senescence, induces senescence and cell death in transformed cells. Similarly, senescence and apoptosis occurred when tumor cells and normal cells were transfected with synthetic poly(I:C) molecules. Activation of the RIG-I/IFIH1/MAVS pathway and de-repression of ERVs were observed in cancer, aging, autoimmune, and neurodegenerative diseases [39]. However, the direct role of ERVs transcripts in maintaining senescence through the RLRs pathway is poorly understood [39, 52, 53].

Although RIG1 and IFIH1 have a very similar structure, they show different binding preferences for dsRNAs, recognizing short (<1 kb) and long (>2 kb) dsRNAs, respectively [54]. In this context, the use of poly(I:C) of different lengths proved that both cytoplasmic dsRNA immunosensors could be efficiently activated during the senescence program, as confirmed by the proper activation of the IFN response. However, we found that compared with RIG1, depletion of IFIH1 and MAVS strongly counteracted the senescence phenotype. The differential biological response of RIG1 and IFIH1 is not clear and requires further investigations but may depend on the altered sensitivity of these cytoplasmic immunosensors to various dsRNA elements [39].

How might the signaling axis ERVs/dsRNAs > IFIH1 > MAVS contribute to senescence? We have found that the strongest effect manifests at the level of SASP; therefore, interfering with the signaling pathway may reduce SASP intensity. Indeed, dsRNAs not only upregulated the expression of type I and type III IFNs but also affected NF-κB-dependent upregulation of proinflammatory genes by turning on MAVS [55, 56]. We confirmed that impairment of MAVS reduced the expression of *IL1B* and *CXCL8*, two known NF-kB targets. Because SASP can mediate enhancement (in an autocrine manner) or induction of senescence (in a paracrine manner) [57], the contribution of ERVs to SASP is a plausible mechanism. However, as SASP is not only involved in enhancing senescence but has other effects [57–59], ERVs activation might also influence other aspects of SASP.

Our results suggest that ERVs upregulated during senescence are not simply passengers of epigenetic reprogramming but contribute to the accumulation of dsRNA species, which play an active role in maintaining the program with important consequences for human aging.

## Materials and methods

### Cell cultures and treatments

BJ-*hTERT*, BJ-*hTERT/HRASG12V*, BJ-*hTERT/myrAKT1*, IMR90, SK-LMS-1, SK-LMS-1^*HDAC4-/-HDAC4PAM-ER*^, were previously described [8]. Parental BJ, IMR90 and SK-LMS-1 have been authenticated by STR DNA Profiling (MWG Eurofins, Germany). BJ-TERT fibroblasts were grown in Earle’s salts minimal essential medium (Euroclone, Milan, Italy) completed with nonessential amino acids (Sigma-Aldrich St. Louis, MO, USA). IMR90 and SK-LMS-1 cells were cultured in Dulbecco modified Eagle medium (Euroclone, Milan, Italy). All mediums were supplemented with 10% of fetal bovine (FBS), L-glutamine 2 mM; penicillin 100 U/mL, and streptomycin, 100 μg/mL; (Euroclone). All cell lines were routinely tested from Mycoplasma contamination.

### Chemical compounds and antibodies

The following chemicals were used: 4-hydroxytamoxifen (4OHT), Doxycycline, Trypan Blue, Aphidicolin, Doxorubicin, BrdU (Sigma-Aldrich, Germany), Camptothecin (Enzo Life Sciences Farmingdale, NY USA), poly (I:C)-HMW/-LMW (InvivoGen, Toulouse, France). Antibodies used are provided in Table S5.

### Immunofluorescence and immunoblotting

For intracellular staining of dsRNA, cells were fixed with 4% paraformaldehyde and permeabilized for 5′ with 0.5% Triton X-100. ICC blocking solution, 3% w/v BSA (Sigma-Aldrich), 3% v/v goat serum (Abcam, Cambridge UK), 0.02% v/v Tween-20 in PBS, was applied for 1 h. Next, incubation with 250 ng J2 antibody in blocking solution was performed. The secondary antibody was Alexa Fluor 546-conjugated (Thermo Fisher Scientific, Waltham, MA USA). Nuclei were stained with Hoechst 33258 (Sigma-Aldrich). Cells were imaged with a confocal microscope Leica AOBS SP8.

For S phase analysis, cells were grown for 3 h with 50 μM BrdU. Cells were fixed with 3% paraformaldehyde and permeabilized with 0.3% Triton X-100. After fixation, coverslips were treated with HCl (2%), quenched with Borate, and processed for immunofluorescence.

Cell lysates after SDS-PAGE and immunoblotting on nitrocellulose were incubated with primary antibodies. HPR-conjugated secondary antibodies were obtained from Cell Signaling Technology (Danvers, Massachusetts, USA) and blots were developed with Super Signal West Dura (Thermo Fisher Scientific).

### dsRNA immunoprecipitation

Total RNA was extracted with Tri Reagent (Molecular Research Center, Cincinnati, OH, USA) from a pellet of 35*10^6^ SKLMS-1 HDAC4 KO cells. IP was performed O/N in Polysomal Lysis Buffer in the presence of 10 μg of J2 antibody. 50 μl slurry protein A (GE Healthcare Chicago, Il, USA) was added and incubated in continuous rotation at 4 °C for 4 h. After 5 washes, RNA was recovered by phenolchloroform extraction/ethanol precipitation and resuspended in 20 μl RNase-free water. The precipitation was repeated until a final amount of 1.6 μg of purified RNA was reached. 800 ng were treated for 20’ at 37 °C with 50U ShortCut RNAse III (NEB, Ipswich, MA, USA), in the digestion buffer supplied by the manufacturer. The digestion was stopped with EDTA. The remaining 800 ng were treated in the same manner but without the addition of RNAse III. 30 pmoles of purified dsRNA treated or not with RNAse III were transfected in recipient cells, by using 15 μl of Lipofectamine 3000 and 250 μl Optimem (Thermo Fisher Scientific, USA).

### dsRNA-seq and data analysis

Total RNA was extracted with Tri Reagent (Molecular Research Center) from a pellet of 35*10^6^ SK-LMS-1*HDAC4*^*-/-/HDAC4PAM-ER*^ cells grown for 96 h with or without 4OHT. Three biological replicates were harvested and subjected to two independent IP. IP was performed O/N in Polysomal Lysis Buffer in the presence of 3 μg of J2 antibody (Scicons, Hungary). The immune complexes were purified by incubating (O/N) each sample with 16 μl of magnetic Dynabeads coated with protein A/G (ThermoFisher). After washing the collected immune complexes three times, RNA was recovered by phenol chloroform extraction/ethanol precipitation and resuspended in 10 μl RNase-free water (Ambion, USA). Concentration, integrity and purity of RNA were checked using Agilent 2100 Bioanalyzer (Agilent RNA 6000 Nano Kit). 10 ng of RNA was digested with DNAseI (NEB), freed from rRNA (using the ribo-zero method) and reverse transcribed to obtain cDNA. Adapters were ligated to the fragmented cDNA after end repair and A-tailing according to the DNBSEQ protocol (MGI-Tech). 18 rounds of PCR amplification were performed to enrich the cDNA fragments. The PCR products were then purified using Ampure XP beads (Agencourt). ssCirDNA was obtained after denaturation and circularization. DNA spheres were generated from each library according to BGI specifications (SOP-SS-025) and subjected to paired-end 100 sequencing at the BGI Genomics facility (BGI Genomics, China). The quality of the reads was assessed using fastqc. The expression levels of different consensus sequences of transposable elements (TEs) were calculated using TEspeX [60]. Reads were mapped to a reference transcriptome using STAR, which consists of TE consensus sequences downloaded from Dfam and the Ensembl transcript sequences containing all coding and non-coding annotated transcripts [61]. To limit the effects of TE fragments embedded in canonical coding and noncoding non-TEs transcripts and to allow quantification of autonomously expressed TEs, reads mapped to TE fragments embedded in coding and/or long noncoding transcripts were discarded. Finally, the number of reads in each TE consensus in each sample was counted to create a consensus sample count matrix. PCA was performed on variance-stabilized transformed read counts by using the varianceStabilizingTransformation function as implemented in the DESeq2 R package [62].

Differentially enriched TE consensus sequences were identified with the edgeR package using the workflow developed by Gao and colleagues [63]. Briefly, the number of reads mapped into each consensus was normalized using the TMM method implemented in edgeR. Then, the dsRIP samples were compared with the corresponding INPUT samples to identify TE in the dsRNA conformation using the generalized linear models (glmQLFTest function) implemented in edgeR with the following formula “(dsRIP+4OHT + dsRIP-4OHT) - (INPUT + 4OHT + INPUT-4OHT)”. Significant results were then identified as TE consensus with a *p* < 0.30 and a |log2FoldChange dsRIP / INPUT | > 0.585.

To identify dsRNA conformational changes between the two tested conditions by normalizing for INPUT samples, we then fitted a generalized linear model, as implemented in the edgeR glmQLFTest function, with the following formula “(dsRIP+4OHT + dsRIP-4OHT) - (INPUT + 4OHT + INPUT-4OHT)”. dsRNA-enriched TEs families were selected as families with a *p* < 0.05, while enriched entities in the ds RIP-4OHT sample were identified with respect to the INPUT + 4OHT sample with a cut-off of |log2FoldChange dsRIP+4OHT / INPUT-4OHT | > 0.585 and |log2FoldChange dsRIP+4OHT /dsRIP-4OHT | > 0.585.

### SA-β-gal assay

Cells were fixed for 5′(PBS 2% formaldehyde/0.2% glutaraldehyde), washed twice and stained for 16 h at 37 °C with staining solution: 40 mM citric acid/Na phosphate buffer, 5 mM K_4_[Fe(CN)_6_]3H_2_O, 5 mM K_3_[Fe(CN)_6_], 150 mM sodium chloride, 2 mM magnesium chloride and 1 mg/mL X-gal (PanReac Applichem, Darmstadt, Germany). Images were acquired with Leica LD bright field optical microscope.

### RNA extraction and quantitative qRT-PCR

A total of 1 μg of total RNA was retrotranscribed using 100 U of M-MLV reverse transcriptase (Invitrogen). qRT-PCR analyses were performed using Bio-Rad CFX96 and SYBR green technology (Sigma-Aldrich). The data were analysed by a comparative threshold cycle using the glyceraldehyde-3-phosphate dehydrogenase (*GAPDH*) and hypoxanthine phosphoribosyltransferase 1 (*HPRT*) as normalizer genes. Oligonucleotides are described in Fig. S6 and Table S6.

### H3K27me3 ChIP-seq experiments and data analysis

ChIP-seq were performed previously [8]. We used the FastQC (v0.11.8) and MultiQC (v1.7) programs to evaluate the quality of the sequencing reads. Bowtie 2 (v2.3.4.3) was used to align reads to the NCBI GRCh38 human genome reference. We performed peak calling of the H3K27me3 and H3K27ac histone marks against inputs using MACS2 (v2.1.2 - broad option, other options as default). Peak heatmaps and profiles were generated using the deepTools (v3.1.3) suite and the gplots (v3.0.1.1), biomaRt (v2.38.0) and Gviz (v 1.24.0) packages, working with R/Bioconductor as previously described [64]. We identified regions showing a consistent variation of the H3K27me3 mark, using Fc > 2 as a cutoff; overlaps of at least one nucleotide between these regions and ERVs were found using the bedtools (v2.28.0) toolset [8].

### Infections, transfections and silencing

Retroviral infections were performed as previously described [65]. For poly (I:C) and RNAi oligos transfections (Table S6), 24 h after plating cells were incubated with OptiMem medium containing Lipofectamine (Thermo Fisher Scientific).

### Statistics

For experimental data, Student *t*-test was employed. Mann–Whitney test was applied when normality could not be assumed. For comparisons between more than two samples, Anova test was applied coupled to Kruskal–Wallis and Dunn’s Multiple Comparison Test. Excel and GraphPad Prism were used for statistical analysis. We marked with *p < 0.05, **p < 0.01, ***p < 0.001.

### Supplementary information

Supplemental figures

Table S1

Table S2

Table S3

Table S4

Table S5

Table S6

Original Data File

Reproducibility checklist

## Data Availability

Raw data corresponding to ChIP-seq experiments are uploaded with GEO accession GSE149644. Raw data corresponding to dsRNA-seq are uploaded with GEO accession GSE250348, https://www.ncbi.nlm.nih.gov/geo/query/acc.cgi?acc=GSE250348. Processed data are available as supplementary tables.
