# Supplementary material for: Transcription of endogenous retroviruses in senescent cells contributes to the accumulation of double-stranded RNAs that trigger an anti-viral response that reinforces senescence
Source: Cell Death Dis. 2024 Feb 21;15(2):157. doi: 10.1038/s41419-024-06548-2 (PMC10882003; doi:10.1038/s41419-024-06548-2)
Supplement: Supplementary file 1 — Supplemental figures [file 41419_2024_6548_MOESM1_ESM.pdf]

Transcription of endogenous retroviruses in senescent cells contributes to the accumulation of double-stranded RNAs that trigger an anti-viral response that reinforces senescence.

Eros Di Giorgio, Liliana Ranzino, Vanessa Tolotto, Emiliano Dalla, Matteo Burelli, Nicolò Gualandi, Claudio Brancolini

SUPPLEMENTAL MATERIALS

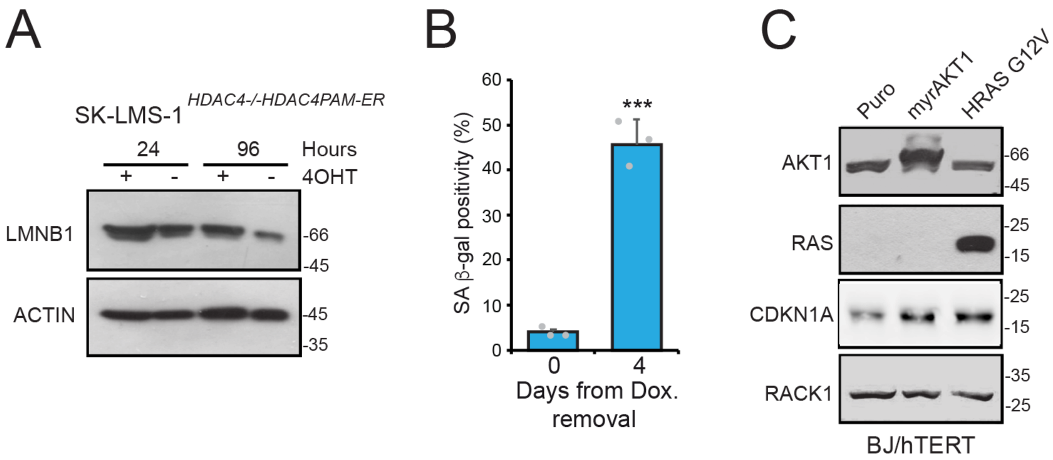

Supplemental Figure S1 related to Figures 1 and 2.

A) Immunoblot analysis using the indicated antibodies. SK-LMS-1 cells knocked-out for HDAC4 and re-expressing a 4OHT inducible, PAM mutated version of HDAC4 (SK-LMS-1$^{HDAC4-/-/HDAC4PAM-ER}$) were grown for the indicated hours in presence or absence of 4OHT.

B) SA β-gal positivity in A375 melanoma cells knocked out for HDAC4 and re-expressing a Dox inducible, PAM mutated version of HDAC4. Cells were grown for 4 days after Dox removal from the medium. Mean ± SD; n =3. *$p < 0.05$, **$p < 0.01$, ***$p < 0.001$, t-test relative to 0.

C) Immunoblot analysis using the indicated antibodies. HRAS G12V and myr-AKT1 were induced for 8 days. CDKN1A levels were used as a marker of senescence induction. RACK1 was used as loading control.

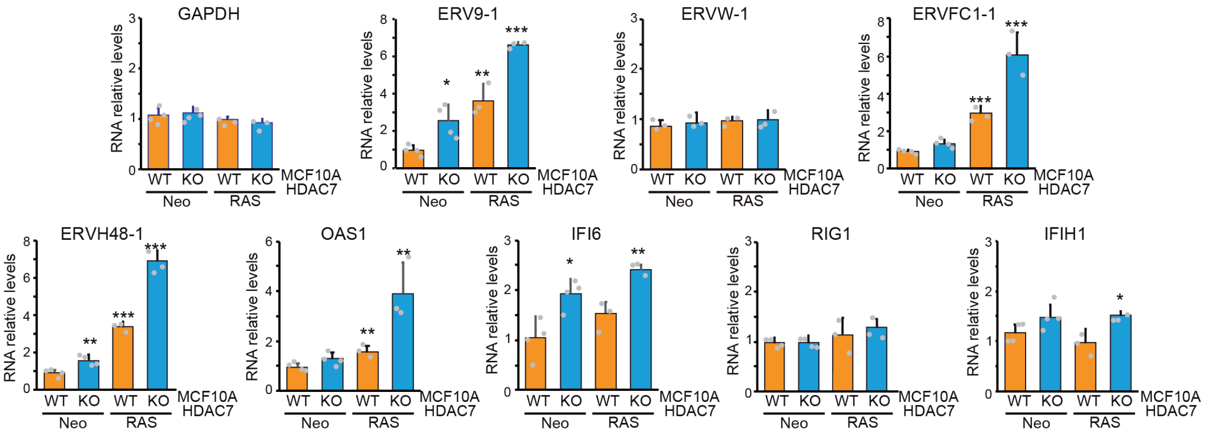

Supplemental Figure S2 related to Figure 2.

Expression levels of the indicated ERVs and ISGs in MCF10A breast epithelial cells knocked out for *HDAC4* and expressing *HRAS* oncogene or *Neo* as control. RNAs were extracted and processed for qRT-PCR. Data are relative to MCF10A-Neo cells expressing HDAC7. Mean ± SD; n = 3 or 4. *p< 0.05, **p< 0.01, ***p< 0.001, t-test relative to WT Neo.

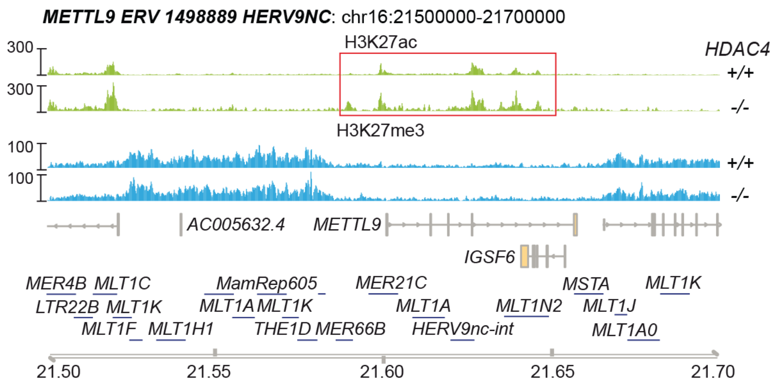

Supplemental Figure S3 related to Figure 3.

Detailed view of H3K27ac (green) and H3K27me3 (light-blue) tracks at a ERVs rich region on Chr16). Regions H3K27 hyper-acetylated in SK-LMS-1/*HDAC4*$^{-/-}$ in respect to the wt are indicated.

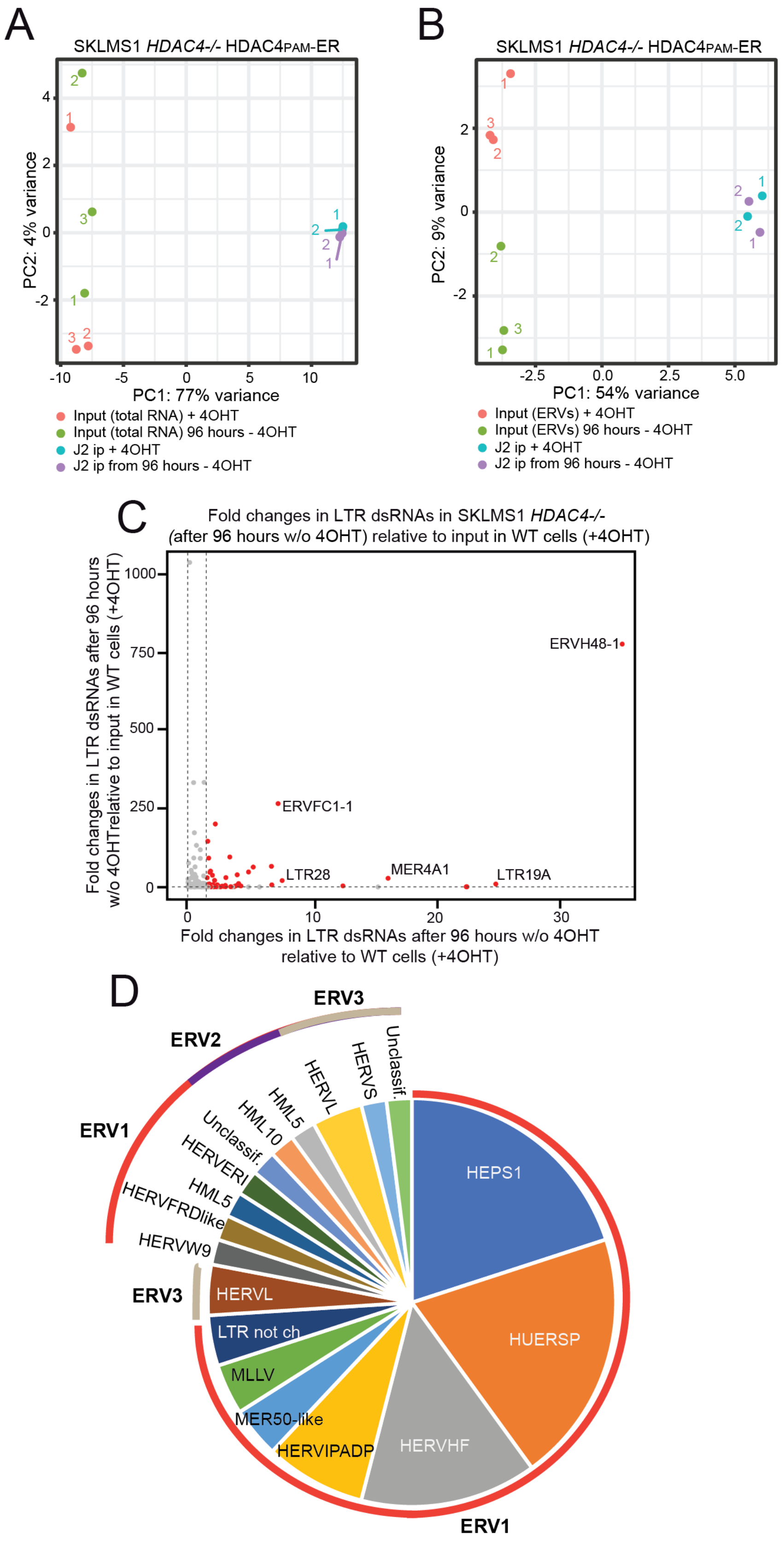

Supplemental Figure S4 related to Figure 5.

A) PCA analysis performed on the input and J2 immunoprecipitated dsRNAs.

B) PCA analysis performed on the input, (only ERVs) and J2 immunoprecipated dsRNAs (only ERVs).

C) Volcano plot that shows the fold changes of LTRs in dsRNA-seq samples obtained from senescent SK-LMS-1$^{HDAC4-/-/HDAC4PAM-ER}$, grown in the absence of 4OHT for 4 days, in respect to proliferating cells, grown in the presence of 4OHT (x axis), and the fold changes of of LTRs in dsRNA-seq samples obtained from SK-LMS-1$^{HDAC4-/-/HDAC4PAM-ER}$ grown in the absence of 4OHT for 4 days, in respect to input from cell grown in the presence of 4OHT (y axis).

D) ERVs classes enriched as dsRNA in senescent SK-LMS-1$^{HDAC4-/-/HDAC4PAM-ER}$ grown in the absence of 4OHT for 4 days, in respect to cells grown in the presence of 4OHT.

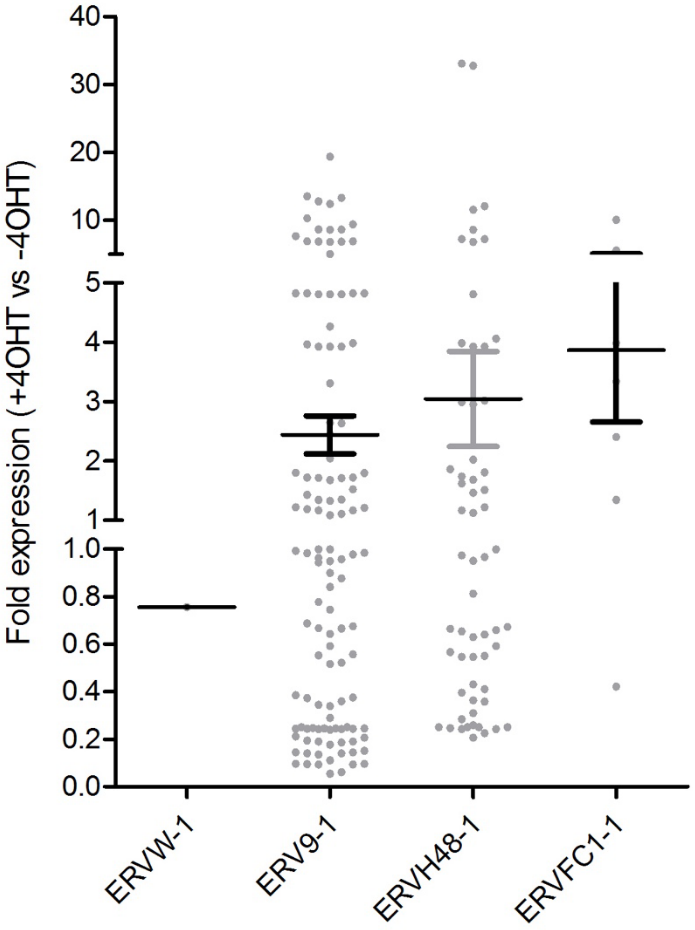

Supplemental Figure S5 related to Figure 5.

Dot plot representing the expression of each single entity belonging to the indicated ERV element in dsRNA-seq preparations obtained from SK-LMS-1$^{HDAC4-/-/HDAC4PAM-ER}$ cells depleted of HDAC4 for 4 days (-4OHT) or expressing HDAC4 (+4OHT).

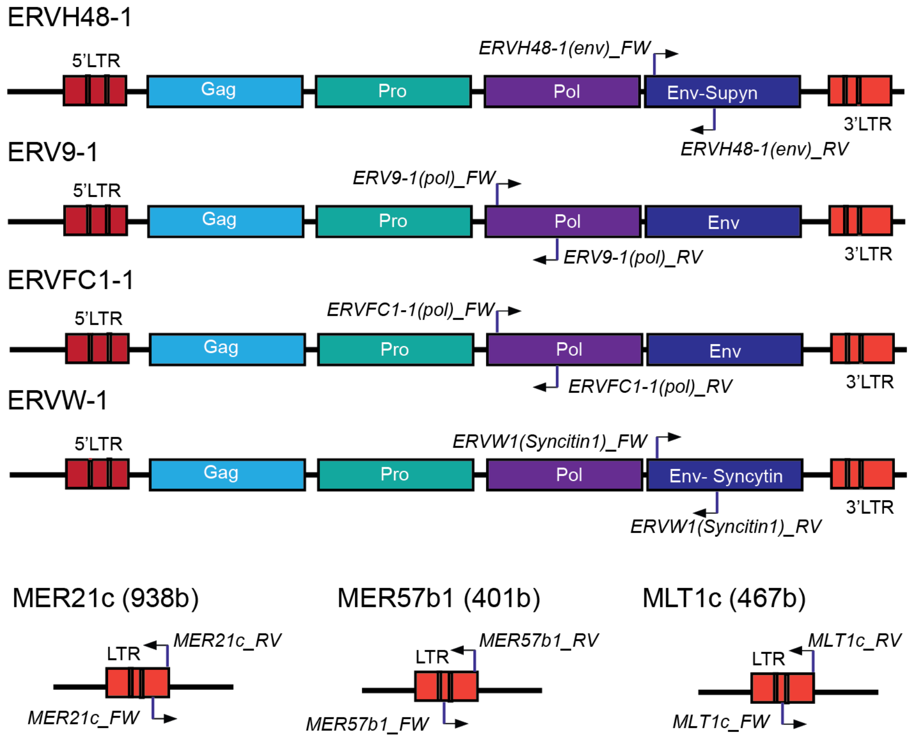

Supplemental Figure S6.
Oligonucleotides used to monitor ERVs expression. The positions of the oligonucleotides are indicated by arrows.

Table S1. Main features of the pharmacologically induced ERVs selected for the study.

Table S2. List of the intergenic ERVs that show a >2 fold decrease in H3K27me3 after depletion of HDAC4 in SK-LMS-1 cells.

Table S3. ERVs enriched in dsRNAs immunopurified from senescent cells after HDAC4 knock-out compared to WT cells.

Table S4. dsRNA species immunopurified from senescent cells after HDAC4 knock-out. Fold changes are relative to input RNA from WT cells.

Table S5. Antibodies used in this study.

Table S6. Oligonucleotides used in this study.
