# Supplementary material for: Transcription of endogenous retroviruses in senescent cells contributes to the accumulation of double-stranded RNAs that trigger an anti-viral response that reinforces senescence
Source: Cell Death Dis. 2024 Feb 21;15(2):157. doi: 10.1038/s41419-024-06548-2 (PMC10882003; doi:10.1038/s41419-024-06548-2)
Supplement: Supplementary file 8 — Original Data File [file 41419_2024_6548_MOESM8_ESM.pptx]

## Slide 1
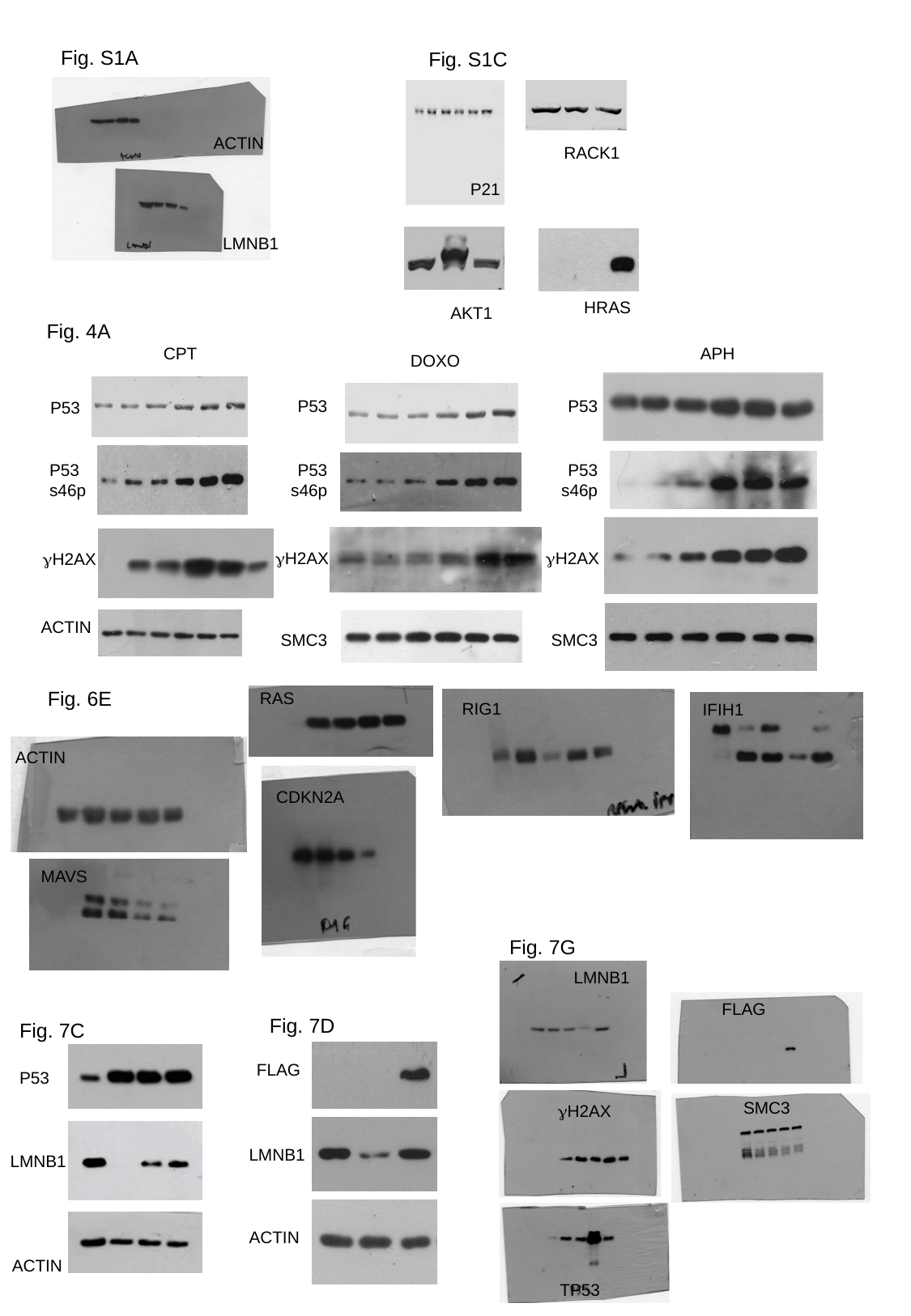

Fig. S1A
Fig. S1C
ACTIN
RACK1
P21
LMNB1
HRAS
AKT1
Fig. 4A
CPT
APH
DOXO
P53
P53
P53
P53 s46p
P53 s46p
P53 s46p
gH2AX
gH2AX
gH2AX
ACTIN
SMC3
SMC3
Fig. 6E
RAS
RIG1
IFIH1
ACTIN
CDKN2A
MAVS
Fig. 7G
LMNB1
FLAG
Fig. 7D
Fig. 7C
FLAG
P53
SMC3
gH2AX
LMNB1
LMNB1
ACTIN
ACTIN
TP53
